# Effects of controlled fermentation by *Lactiplantibacillus plantarum* PBS067 on *Vitis vinifera* L. leaf extract

**DOI:** 10.1007/s00253-026-13942-7

**Published:** 2026-06-29

**Authors:** Marta Armari, Federica Cascella, Aurora Tritto, Farah Hamed, Shwetha Bangalore Renukaprasad, Ana Moreno Villena, Giuseppe Meca De Caro, Carlos Luz Mínguez, Cesar Francisco Trejo, Barbara Azzimonti

**Affiliations:** 1 Department of Health Sciences, School of Medicine, Università del Piemonte Orientale, 28100 Novara, Italy; 2 Center for Translational Research On Autoimmune and Allergic Diseases, Università del Piemonte Orientale, 28100 Novara, Italy; 3 Roelmi HPC Srl, Via Celeste Milani 24/26, 21040 Origgio, Varese, Italy; 4 Laboratory of Food Chemistry and Toxicology, Faculty of Pharmacy, University of Valencia, Av. Vicent Andrés Estellés S/N, 46100 Burjassot, Spain; 5 Centro 3R - Centro Interuniversitario Per La Promozione Dei Principi Delle 3R Nella Didattica E Nella Ricerca, Pisa, Italy

**Keywords:** *Lactiplantibacillus plantarum* PBS067, Probiotic-driven fermentation, *Vitis vinifera* L. leaves, Waste valorization

## Abstract

**Abstract:**

*Vitis vinifera* L. leaves represent one of the most phenolic-rich grapevine biomasses, exhibiting a broad spectrum of biological activities, including prebiotic and microbiota-modulating ones. However, as they are mainly regarded as agro-industrial waste, they are still an underexploited resource. Probiotic-driven fermentation may offer a promising strategy to enhance their bioactive potential while addressing this challenge. The aim of this study was to evaluate the effects of the in vitro fermentation of *Vitis vinifera* L. leaf extract (VVLE) by *Lactiplantibacillus plantarum* PBS067 in terms of phenolic content, antioxidant activity, lactic acid and protein content, and metabolomic profile. Our analysis shows the prebiotic activity of VVLE, as VVLE-supplemented medium allowed probiotic growth without toxic effects. Leaves fermentation led to a significant enrichment in lactic acid and the biotransformation of complex plant-secondary metabolites into smaller derivatives. However, VVLE antioxidant capacity slightly decreased after the fermentation, likely due to the phenolic conversion itself. These results highlight how controlled lacto-fermentation valorizes grapevine leaves, transforming them into sustainable upcycled bioactive products with potential applications within cosmetics, pharmaceuticals, nutraceuticals and complementary medical product sectors.

**Key points:**

• *L. plantarum PBS067 thrives in medium supplemented with polyphenol-rich leaf extract*

• *L. plantarum PBS067-driven fermentation modulates VVLE phytochemical profile*

• *L. plantarum PBS067-driven fermentation valorizes viticulture by-products*

**Graphical abstract:**

**Figure:**
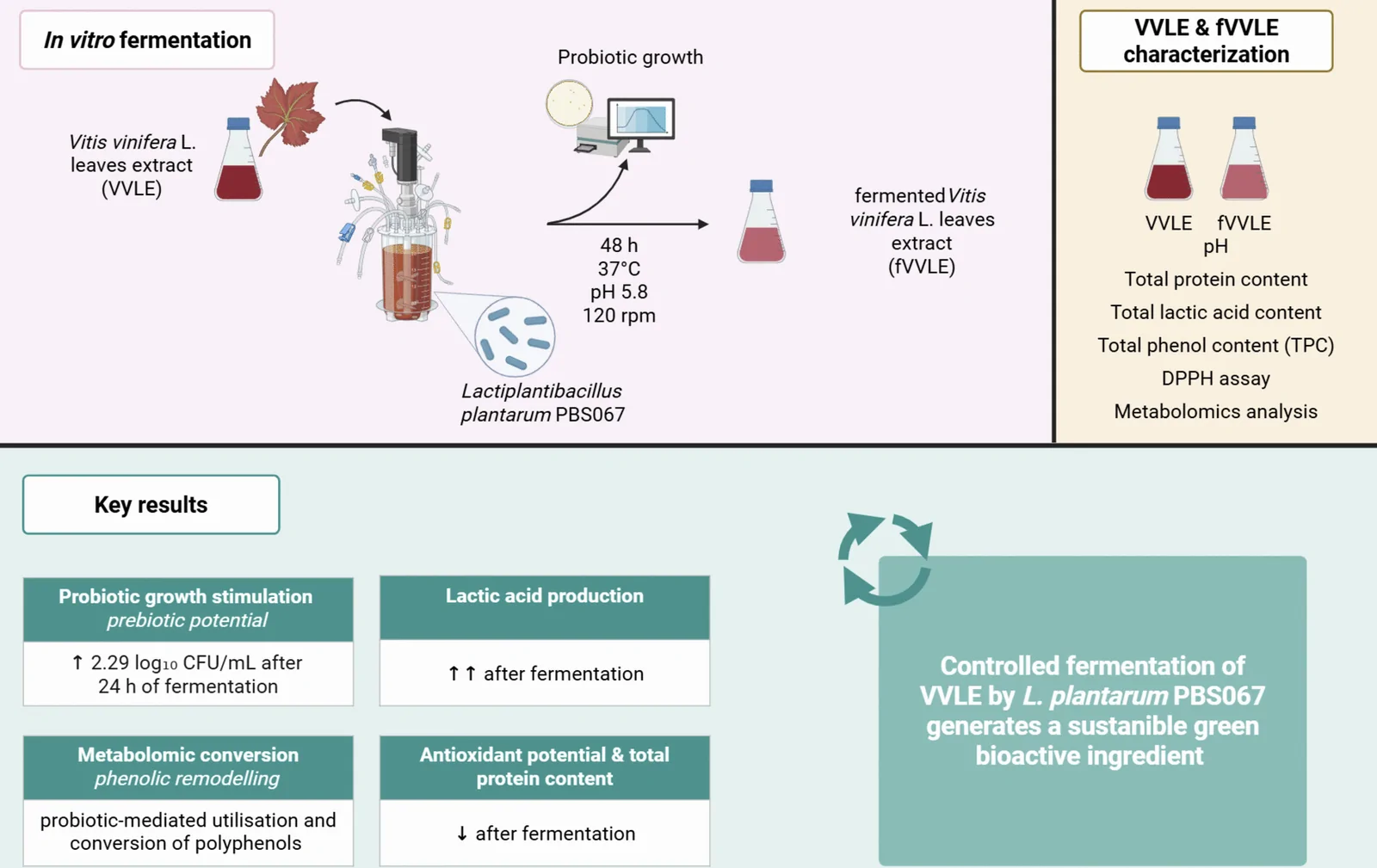

**Supplementary Information:**

The online version contains supplementary material available at https://doi.org/10.1007/s00253-026-13942-7.

## Introduction

Viticulture is one of the most ancient and recognizable agricultural practices adopted on a global scale. It is well established that it holds significant economic, cultural, and ecological value across European countries and the Mediterranean basins (Aouey et al. 2016; Rouxinol et al. 2023). *Vitis vinifera* L., the most widely cultivated grapevine species, thrives across temperate and subtropical regions of the northern hemisphere and comprises up to 5000 true cultivars (Gonçalves et al. 2024). Although these varieties are also used for fresh consumption, as dried fruit, and distilled liquor, their main application is within the wine industry (Ali et al. 2010; Goufo et al. 2020).Throughout the winemaking process, several by-products are obtained from the grapes, including leaves, skin, pulp, and seeds (Serra et al. 2023). These biomasses are natural sources of plant secondary metabolites (PSMs), including phenolic acids, flavonoids, and stilbenes, which are commonly classified in flavonoid and non-flavonoid compounds based on their carbon skeleton (Armari et al. 2024; Perestrelo et al. 2014). The most prevalent flavonoids found in grapevine by-products include flavonols (e.g., quercetin and kaempferol, as well as their glycosylated derivatives), flavan-3-ols (e.g., catechin and epicatechin), and anthocyanins (e.g., cyanidin and malvidin) (Armari et al. 2024). Meanwhile, non-flavonoids mainly comprise phenolic acids (e.g., hydroxycinnamic and hydroxybenzoic acids) and stilbenes (e.g., resveratrol and its oligomeric forms) (Chedea et al. 2021; El Rayess et al. 2024; Goufo et al. 2020). Evidence suggests that these bioactive compounds possess a broad spectrum of health-promoting activities, including antioxidant, anti-inflammatory, immunomodulatory, prebiotic, and antimicrobial properties, with promising applications in the cosmetic, nutraceutical and pharmacological sectors (Castro et al. 2024; Fernandes et al. 2013; Nassiri-Asl and Hosseinzadeh 2009).

Notably, different wine industry by-products have a differential composition in phenolic compound classes (Silva et al. 2023). The phytochemical content of grape pomace extracts is mainly represented by phenolic acids, flavonoids, tannins, and stilbenes (de la Cerda-Carrasco et al. 2015). Grape skin from red cultivars is a rich source of anthocyanins, accounting for its antioxidant activity and potential cardioprotective effect (Kallithraka et al. 2009). Seed extracts have been reported to contain a great amount of proanthocyanidins (PACs), making them valuable for applications targeting antioxidant and anti-inflammatory activities (Rodríguez-Pérez et al. 2019). Grapevine leaves are distinguished by their high phenolic content, mainly due to the presence of quercetin, kaempferol and catechin, often found in their glycosylated or esterified forms. Similarly, the non-flavonoid fraction also actively contributes to the total polyphenol content (Lacerda et al. 2016; Meng et al. 2021). Hydroxybenzoic and hydroxycinnamic acids are among the main phenolic acids found in grape leaves, widely known for their antioxidant, antidiabetic, anti-inflammatory, antibacterial, antifungal, antiviral, microbiota-modulating, anti-obesity, hepato- and gastro-protective activities (Singh et al. 2023). Moreover, in the skin care field, several hydroxycinnamic derivatives, such as caffeic and ferulic acids, have been reported to have skin-whitening effects through the inhibition of tyrosinase activity and the modulation of melanogenesis-related pathways (Kim et al. 2025; Zduńska et al. 2018).

Like any other by-product, grape leaves are normally discarded as solid waste of the winemaking process (Baroi et al. 2022), with detrimental effects on the environment such as water pollution, soil degradation and greenhouse gas emissions (Serra et al. 2023). However, emerging trends within the green circular economy model have provided a more sustainable alternative for their recovery and management (Hoss et al. 2021; Pantelic et al. 2023). Among the most promising strategies for the commercial valorization of grapevine by-products, microbial fermentation offers sustainable solutions for many industries, enhancing the bioactivity and bioavailability of phenolic compounds (Pérez-Rivero and López-Gómez 2023). Precise fermentation requires starter cultures of bacteria, yeasts, and/or molds (Khayatan et al. 2024; Mathur et al. 2020; Sionek et al. 2023; Yassunaka Hata et al. 2023). Many factors play a critical role in the choice of the best starter for every bioprocess, as these microorganisms are responsible for the production of various metabolites (e.g., acids, alcohol, vitamins, peptides, aldehydes, and smaller phenolic compounds), which actively contribute to the unique characteristics of the final fermented product (Sawant et al. 2025). Lactic acid bacteria (LAB) are a diverse group of Gram-positive bacteria commonly used as starters in the fermentation process due to their ability to produce organic acids, especially lactic acid, exopolysaccharides, and other substances from carbohydrate sources (Khayatan et al. 2024; Yang et al. 2024). LAB have been shown to possess a wide variety of health-promoting properties, since they can modulate gastrointestinal health, enhance hepatic activity, scavenge free radicals, as well as reduce cholesterol levels and blood pressure (Kuo et al. 2021; Yeo and Ewe 2015). The genus *Lactobacillus* comprises more than 300 valid species and is undoubtedly the most prominent and heterogeneous group of LAB (Zheng et al. 2020). Among them, *Lactiplantibacillus plantarum* (formerly *Lactobacillus plantarum*) possesses proven probiotic potential, metabolic flexibility, and safety profile (Katiku et al. 2022). Notably, its remarkable enzymatic ability to convert phenolic compounds in natural extracts and unprocessed food into derivatives with novel biofunctional properties has found wide applications in industrial fermentation processes (Behera et al. 2018; Echegaray et al. 2023).

Given its chemical diversity, *V. vinifera* leaves represent a highly promising substrate for microbial fermentation. For instance, its total phenol and flavonoid contents provide an optimal biochemical environment for microbial enzymes to process, allowing for the biotransformation of compounds with improved solubility, bioavailability, and functionality. This makes grapevine leaves technically suitable for controlled fermentation processes aimed at producing high-value bioactive ingredients. While grape pomace (De Bellis et al. 2022), skin (Liu et al. 2019), and juice (Mirmohammadi et al. 2021) have been previously investigated as fermentation substrates for *L. plantarum*, the use of *V. vinifera* L. leaf extract (VVLE) has received limited attention. This gap in knowledge is particularly relevant considering the growing demand for novel upcycled and sustainable bioactive compounds, as well as more effective management of agricultural waste for industrial applications (e.g., cosmetics, pharmaceuticals, nutraceuticals, and complementary medical products).

The primary aim of this study is to investigate the impact of fermentation by *L. plantarum* PBS067 on the biochemical and metabolic profile of VVLE under controlled and pilot-scale conditions. Specifically, the study evaluates the changes induced by probiotic-mediated fermentation in the phenolic composition, antioxidant capacity, metabolomic profile, lactic acid production, and protein content of VVLE before and after fermentation. By characterizing these fermentation-driven modifications, this work seeks to provide insights into the potential valorization of grapevine leaves as a sustainable source of bioactive ingredients and to support their future industrial application within an upcycled framework.

## Materials and methods

### Plant material and extract preparation

Leaves of *Vitis vinifera* L. cultivar *rubra* were harvested from selected vineyards from the Mediterranean area and immediately processed by INCOS-Cosmeceutica Industriale (Funo di Argelato, Bologna, Italy). Extraction was performed by maceration of the air-dried and crushed vine leaves in sterile double-distilled water (ddH_2_O) at 60 °C using a proprietary procedure designed to preserve the phenolic content. Next, VVLE was filtered with a 200 μm nylon filter, then refrigerated, and refiltered with a 50 μm nylon filter. At the end of the process, the product was packaged in 10-L tanks and stored at room temperature (RT).

### Bacterial strain and growth conditions

*Lactiplantibacillus plantarum* PBS067 (DSM 24937, Leibniz Institute DSMZ, German Collection of Microorganisms and Cell Cultures, GmbH, Germany), provided by Synbalance Srl for ROELMI HPC Srl (Origgio, Varese, Italy), was used to conduct the VVLE fermentation experiments below. The bacterial strain was stored in glycerol stocks at −80 °C until use. To prepare *L. plantarum* PBS 067 working cultures, an aliquot of one glycerol stock was streaked onto Man, Rogosa & Sharpe (MRS, VWR® International Srl, Milan, Italy) agar plates and aerobically incubated for 48 h (h). Then, single colonies were picked and transferred each into fresh MRS broth and incubated overnight (ON) in static aerobic conditions. The strain was routinely renewed as described above.

### Inoculum preparation and upstream fermentation process

Fresh ON *L. plantarum* PBS067 culture was adjusted to approximately 1 × 10^8^ colony forming units (CFU)/mL prior to inoculation at 2% v/v into the fermentation medium. Probiotic-driven fermentation was carried out in a batch bioreactor in Poly I medium (patent pending, ROELMI HPC Srl) supplemented with VVLE, previously sterilized using 0.22 μm polyvinylidene difluoride (PVDF) filters (VWR® International Srl, Italy). Fermentation parameters were optimized based on preliminary experiments, through which the optimal bacterial growth conditions were selected. The fermentation was conducted in a 1-L Applikon autoclavable MiniBio Reactor System (Applikon® Biotechnology B.V., Delft, The Netherlands), with continuous control, for up to 48 h, of temperature (37 °C ± 0.1), agitation (120 rpm), and pH (5.8 ± 0.1) by means of a my-Control bio controller (Applikon® Biotechnology B.V., Delft, The Netherlands). An alkaline base (ammonium 10%), connected to the bioreactor through a pump, enabled to compensate for the decrease in pH generated by the lactic acid produced during fermentation.

### Probiotic growth assessment during VVLE fermentation

The probiotic strain growth was monitored by OD_600_ reading using a Tecan Spark® spectrophotometer (Tecan Trading AG, Mannedorf, Switzerland) right after the inoculation (T_0_), and after 2, 4, 6, 24, and 48 h of the fermentation process. Furthermore, CFU count on MRS-agar plates was performed at the same timepoints to directly measure viable dividing bacteria cells. Samples were serially tenfold diluted in 0.9% sterile saline solution. For each selected dilution, 100 μL were confluence plated onto MRS-agar plates and incubated in aerobic conditions at 37 °C for 48 h. Plates with a colony number between 30 and 300 were considered to determine the CFU/mL with the following formula:

$$\frac{\mathrm{C}\mathrm{F}\mathrm{U}}{\mathrm{m}\mathrm{L}}= \frac{\mathrm{n}\mathrm{u}\mathrm{m}\mathrm{b}\mathrm{e}\mathrm{r} \mathrm{o}\mathrm{f} \mathrm{c}\mathrm{o}\mathrm{l}\mathrm{o}\mathrm{n}\mathrm{i}\mathrm{e}\mathrm{s}*\mathrm{d}\mathrm{i}\mathrm{l}\mathrm{u}\mathrm{t}\mathrm{i}\mathrm{o}\mathrm{n} \mathrm{f}\mathrm{a}\mathrm{c}\mathrm{t}\mathrm{o}\mathrm{r}}{\mathrm{v}\mathrm{o}\mathrm{l}\mathrm{u}\mathrm{m}\mathrm{e} (\mathrm{m}\mathrm{L})}$$

Each measurement was performed in replicates.

### Downstream process and pH measurement

At the end of the fermentation process, the broth was centrifuged at 5000 rpm at 15 °C for 15 min (min). The supernatant was 0.22 μm PVDF filtered, aliquoted, and stored at controlled temperature (−20 °C). The pH of the filter-sterilized VVLE and its fermented product (fVVLE) was also determined (Sension + PH3, Hach Lange S.r.l., Milan, Italy).

### Antioxidant activity assessment

The antioxidant capacity of both VVLE and fVVLE was evaluated by using a modified version of the protocol for the DPPH (2,2-diphenyl-1-picrylhydrazyl) assay described by Gulcin and Alwasel (2023). Briefly, DPPH concentration of 400 μM (μmol/L) was selected according to the regression curve (Supplementary Fig. S1, *y* = 0.0023*x* + 0.1445, *R*^2^ = 0.9977).

Samples were mixed with the DPPH solution in a 1.25:1 ratio and incubated in the dark at RT for 30 min. Subsequently, 150 µL of the solution was transferred into a 96-well plate and 517 nm readings were taken using the Tecan Spark® spectrophotometer. A negative control containing DPPH solution only was also used. Sample antioxidant capacity was expressed as the percentage of inhibition of the radical activity of DPPH (I%) (Bordiga et al. 2015) and calculated using the following formula:

$$I\%= \frac{({Abs}_{\mathrm{c}\mathrm{o}\mathrm{n}\mathrm{t}\mathrm{r}\mathrm{o}\mathrm{l}}-{Abs}_{\mathrm{s}\mathrm{a}\mathrm{m}\mathrm{p}\mathrm{l}\mathrm{e}})}{{Abs}_{\mathrm{c}\mathrm{o}\mathrm{n}\mathrm{t}\mathrm{r}\mathrm{o}\mathrm{l}}}*100$$

The experiment was replicated three times on separate days, with measurements performed in triplicate.

### Total phenolic content (TPC) assay

The total phenolic content (TPC) of VVLE and fVVLE was determined through a slight modification of the Folin-Ciocalteu assay described by Singleton et al. (Singleton et al. 1999). For the experiments, 0.5 mL of each sample was mixed with 2.4 mL of ddH_2_O, 2 mL of 2% w/v sodium carbonate solution (Na_2_CO_3_), and 0.1 mL of Folin-Ciocalteu reagent. After incubation in the dark for 60 min, a volume of 150 µL was transferred into a 96-well plate and absorbance was measured at *λ* = 750 nm. To exclude the possibility of interference from the reagents, a negative control, made by replacing the sample with 0.5 mL of ddH_2_O, was also measured. The total phenolic content was expressed as milligrams of gallic acid equivalents per solution liter (mgGAE/L), by solving the equation of the straight line shown in Supplementary Fig. S2 (*y* = 0.0005*x* + 0.01648, *R*^2^ = 0.9957). The experiment was replicated three times on separate days, with measurements performed in triplicate.

### Metabolomic profiling evaluation

High-performance liquid chromatography (HPLC) was employed to analyze the phenolic composition of VVLE and fVVLE. The analysis focused on the content in major polyphenolic classes and specific marker compounds. Briefly, 1 mL of each sample was diluted with 9 mL of Milli-Q water. Diluted samples were filtered through PTFE membrane filters (0.2 μm), placed into amber LC vials and 5 μL aliquots were injected into the UHPLC-Q-TOF-MS system for analysis. The analysis utilized an UHPLC system (1290 Infinity II LC, Agilent Technologies) comprising an automatic sampler, binary pump, and vacuum degasser, coupled to a quadrupole time-of-flight mass spectrometer (Agilent 6546 LC/Q-TOF) operating in positive and negative ionization mode (Agilent Technologies, Santa Clara, CA, USA). A 5 μL injection volume was applied, with a total run time of 25 min per analysis. Chromatographic separation was performed on an Agilent Zorbax RRHD SB-C18 column (2.1 × 50 mm, 1.8 μm). The mobile phases consisted of Milli-Q water (A) and acetonitrile (B), both supplemented with 0.1% formic acid. The gradient elution program was as follows: 2% B at 0 min, ramped to 95% B at 22 min, and returned to 5% B at 25 min, with a re-equilibration period of 3 min between injections. The flow rate was set at 0.4 mL/min. Source conditions for the Dual AJS ESI were as follows: drying gas temperature 325 °C, drying gas flow 10 L/min, nebulizer pressure 40 psig, sheath gas temperature 295 °C, sheath gas flow 12 L/min, capillary voltage 4000 V, nozzle voltage 500 V, fragmentor voltage 120 V, skimmer voltage 70 V. Data were acquired in the m/z range of 50–1500 Da with a full-scan rate of 5 spectra/s and MS/MS acquisition at 3 spectra/s, allowing a maximum of two precursors per cycle, with collision energies set at 10, 20, and 40 eV.

Targeted/semi-targeted LC/Q-TOF metabolomics workflow was applied to characterize the metabolites contained in samples. Data processing, chromatographic peak integration, and metabolite identification were carried out with MassHunter Qualitative Analysis Software B.08.00 and PCDL Manager B.08.00. Identification criteria included a score threshold of 95% and a mass accuracy tolerance of 1 ppm. For the calibration of the UHPLC-Q-TOF-MS analysis method, commercial standards for the main phenolic acids, including DL-3-phenyllactic acid and 3–4-dihydroxyhydrocinnamic, were used at concentrations of 0.08, 0.4, 2, and 10 mg/L. The LOD was determined using a signal-to-noise ratio of 3:1 and the LOQ a signal-to-noise ratio of 10:1.

A comparison of the relative abundance of each of the metabolites present in the different products has been carried out. Relative abundances and their comparisons were based on normalized chromatographic peak areas obtained under consistent instrumental conditions.

### Total lactic acid content quantification

As LAB convert carbohydrates into organic acids, mainly lactic acid, both the VVLE and the fVVLE were assessed for the concentration of L-(+)- and D-(−)-lactic acid by using the D/L-Lactic Acid Megazyme Assay Kit (NEOGEN Europe Ltd., Ayr, UK). Total lactic acid content was manually determined following the manufacturer’s instructions by measuring the absorbance at *λ* = 340 nm using the NanoPhotometer NP80 (Implen, Munich, Germany). Total lactic acid concentration was expressed as mg/mL and calculated as follows:

$$\mathrm{T}\mathrm{o}\mathrm{t}\mathrm{a}\mathrm{l} \mathrm{l}\mathrm{a}\mathrm{c}\mathrm{t}\mathrm{i}\mathrm{c} \mathrm{a}\mathrm{c}\mathrm{i}\mathrm{d} \left(\frac{\mathrm{m}\mathrm{g}}{\mathrm{m}\mathrm{L}}\right)= \frac{V*MW}{\varepsilon *d*v}*\Delta {A}_{\mathrm{T}\mathrm{o}\mathrm{t}\mathrm{a}\mathrm{l} \mathrm{l}\mathrm{a}\mathrm{c}\mathrm{t}\mathrm{i}\mathrm{c} \mathrm{a}\mathrm{c}\mathrm{i}\mathrm{d}}$$

with *V*, cuvette volume; MW, lactic acid molecular weight; *ε*, molecular extinction coefficient of NADH at 340 nm (6300 L/mol *cm); *d*, cuvette optical path; *v*, sample volume; *ΔA*_Total lactic acid_, absorbance difference.

The experiment was replicated three times on separate days, with each measurement performed in triplicate.

### Total protein content quantification

Both VVLE and fVVLE were assessed for their total protein concentration using the colorimetric-based Micro BCA™ (bicinchoninic assay) Protein Assay (Thermo Scientific™, Waltham, MA, USA), according to the manufacturer’s instructions. Briefly, the samples were plated into a 96-well plate with the BCA working solution. Then, the plate was incubated at 37 °C for 2 h, and the absorbance was read at *λ* = 562 nm. For the determination of the sample protein concentration, expressed in μg/mL, a standard curve was prepared using Bovine Serum Albumin (BSA, Thermo Scientific™, Waltham, MA, USA), within the range of 0.5–200 μg/mL (Supplementary Fig. S3, *y* = 8.9589*x*^2^ + 69.495*x* + 0.6744, *R*^2^ = 1). The experiment was replicated three times on separate days, with all measurements performed in triplicate.

### Statistical analysis

Data were expressed as mean ± standard deviation (SD). Statistical analyses (unpaired *t*-test for gaussian data, Mann-Whitney test for non-gaussian data) were performed using GraphPad Prism version 8.0.1 for Windows (GraphPad Software, San Diego, CA, USA, www.graphpad.com) to identify significant differences (*p* < 0.05).

## Results

### *L. plantarum* PBS067 prebiotic-stimulated growth during the fermentation process

**Fig. 1:**
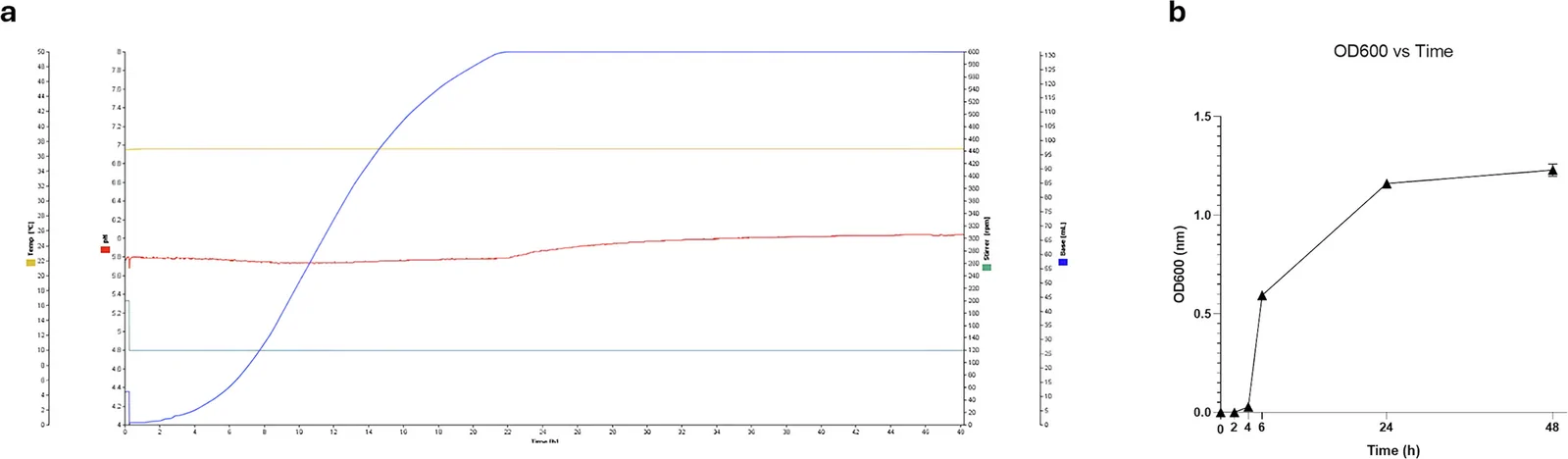
*L. plantarum* PBS067 growth during fermentation. a Real-time monitoring of *L. plantarum* PBS067 fermentation parameters in the bioreactor. Online monitoring of the fermentation process performed in 1-L Applikon MiniBio Reactor System at controlled conditions (temperature 37 °C ± 0.1, pH 5.8 ± 0.1, agitation 120 rpm) for 48 h. Data acquisition was performed using the software Lucullus PIMS Lite/my-Control bio controller system. Different curves represent distinct fermentation parameters tracked during the cultivation process (blue curve: addition of alkaline base; red curve: pH; yellow curve: temperature; green curve: agitation (rpm)). b *L. plantarum* PBS067 growth curve in Poly I medium during VVLE fermentation. OD600 (nm) = optical density at 600 nm. Data are expressed as mean ± SD

In vitro* L. plantarum* PBS067 growth within the bioreactor was indirectly assessed by monitoring the quantity of alkaline base added during the fermentation. Indeed, as reported in literature, lactic acid production associates with *L. plantarum* PBS067 growth and naturally leads to a pH reduction of the fermentation medium (Popova-Krumova et al. 2024). In our system, the medium acidification was compensated by the addition of the alkaline base to maintain the optimal pH, with an increase in its release during the first 22 h, then reaching plateau (Fig. 1a, blue curve).

Concurrently, probiotic growth was assessed by means of OD_600_ readings and CFU/mL counting (Fig. 1b, Supplementary Table S1). During the fermentation process, probiotic cell counts increased by approximately 2.29 log₁₀ CFU/mL, going from 7.83 ± 0.02 log₁₀ CFU/mL at T_0_ to 10.12 ± 0.04 log₁₀ CFU/mL at T_24_ (Supplementary Table S1). The comparison between OD_600_ readings and CFU/mL suggests that the lag phase lasted approximately 4 h, with minimal OD_600_ shifts (0 ± 0.00 at T_0_ and T_2_, 0.03 ± 0.02 at T_4_). At 6 h (T_6_), both the OD_600_ and the cell count increased (0.59 ± 0.00 and 9.32 ± 0.04 log₁₀ CFU/mL, respectively), indicating the start of the exponential phase, while data at 24 h (T_24_) suggested the establishment of the stationary phase (OD_600_ = 1.16 ± 0.00; log_10_ CFU/mL = 10.12 ± 0.04). At 48 h (T_48_), we still observed a high OD_600_ reading (1.23 ± 0.03), but we also recorded a reduction in viable bacteria compared to T_24_ (9.07 ± 0.08 *vs* 10.12 ± 0.04 log_10_ CFU/mL), indicating that *L. plantarum* PBS067 had probably entered the death phase.

Notably, the blue curve in Fig. 1a, indicating the alkaline base addition, closely corresponds to the increase in OD_600_ readings previously reported, demonstrating a clear parallelism between base consumption (or acid production) and bacterial growth kinetics. This curve reaches a plateau around 22 h (T_22_), which aligns with the bacterial growth steady state observed through OD_600_ measurements, confirming that alkaline base monitoring may serve as a reliable indirect assessment of probiotic growth.

### VVLE and fVVLE preliminary characterization

After the end of the fermentation process and consequential filtration, VVLE and fVVLE features were compared (Table 1). The final product has a higher pH value compared to the starting substrate (6.12 ± 0.01 vs 4.76 ± 0.02). Secondly, the total lactic acid content at 340 nm, based on NADH amount formation, was assessed. As expected, the fermented product is much richer in total lactic acid, while the VVLE contains minimal levels (94.76 mg/mL ± 8.55 vs 0.74 mg/mL ± 0.27, *p* < 0.0001). Lastly, the total protein content based on the BCA principle was determined (36.92.31 mg/mL ± 8.73 vs 52.73 mg/mL ± 6.18 for fVVLE and VVLE respectively, *p* = 0.0003).

**Table 1:** Preliminary comparative characterization of VVLE and fVVLE

	**pH**	**Total lactic acid content (mg/mL)**	**Total protein content (g/mL)**
VVLE	4.70 ± 0.05	0.74 ± 0.27	52.73 ± 6.18
fVVLE	6.39 ± 0.04	94.76 ± 8.41	36.92 ± 8.73

**Fig. 2:**
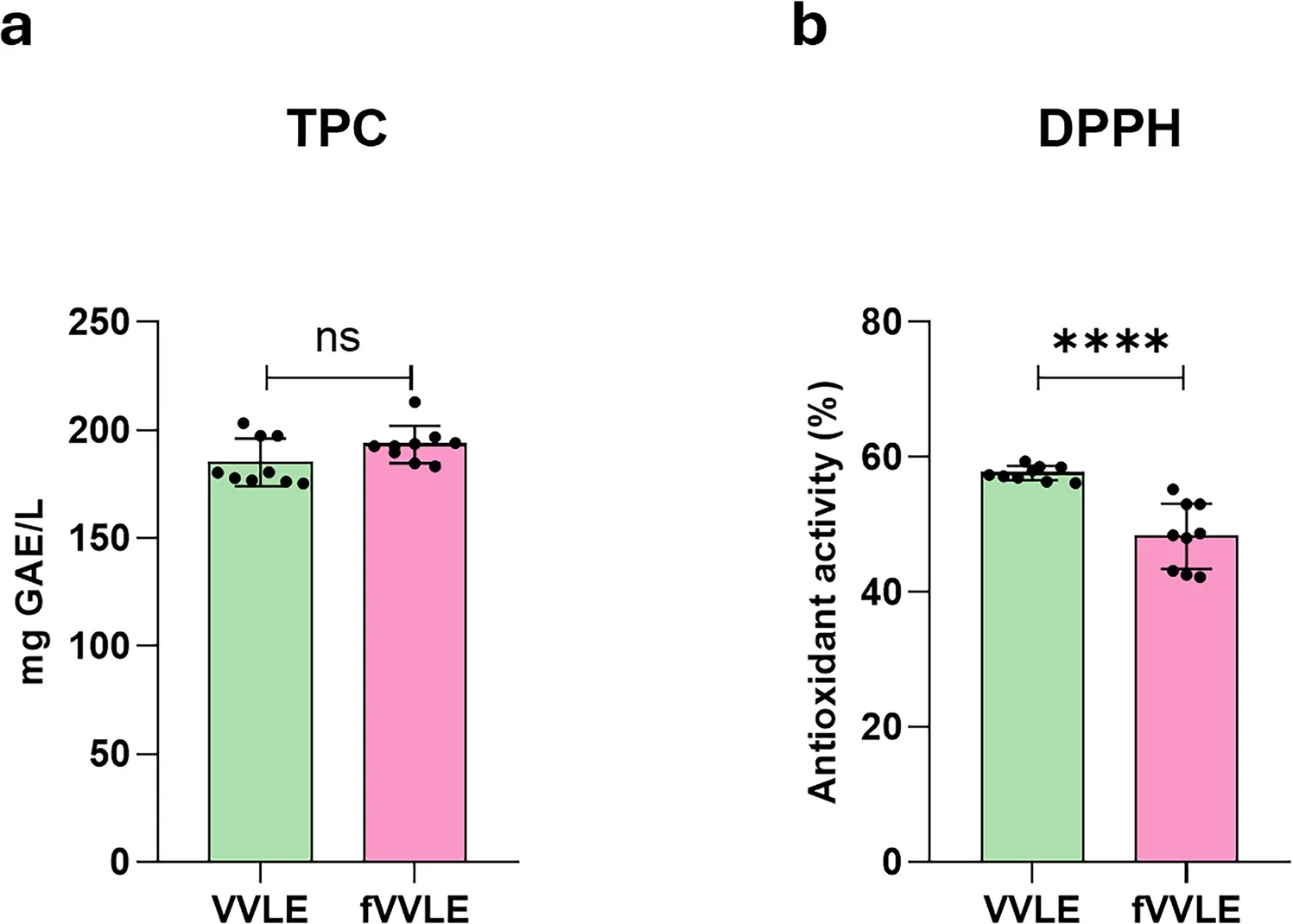
Characterization of VVLE and fVVLE. a Total phenolic content (TPC) in VVLE and fVVLE, measured in mg GAE/L. b 2,2-Diphenyl-1-picrylhydrazyl (DPPH) activity of non-fermented and fermented extract, measured in % through DPPH assay. GAE/L, gallic acids equivalents/liter. VVLE, Vitis vinifera L. leaves extract; fVVLE, fermented Vitis vinifera L. leaves extract. Data are expressed as the mean ± SD. ****p < 0.0001

Phenolic profile comparison between VVLE and fVVLE..TPC was measured before and after 48 h of probiotic-driven fermentation to evaluate the impact of the starter culture on VVLE composition and properties. The Folin-Ciocalteu assay revealed a modest but reproducible rise in the TPC after lactic fermentation (Fig. 2a), ranging from 185.01 ± 11.05 mg GAE/L in VVLE to 193.37 ± 8.59 mg GAE/L in fVVLE (p > 0.05).

### Changes in VVLE antioxidant activity after the fermentation process

The antioxidant effect of VVLE and fVVLE was determined by measuring the in vitro DPPH radical scavenging activity. Grapevine-fermented extract exhibits significantly reduced antioxidant activity compared to the raw extract (from 57.63 ± 1.09 to 48.26 ± 4.85; *p* < 0.0001) (Fig. 2b). Thus, probiotic-guided fermentation process caused a measurable decline in antioxidant capacity (%) of the leaf extract.

### Metabolomics analysis

**Fig. 3:**
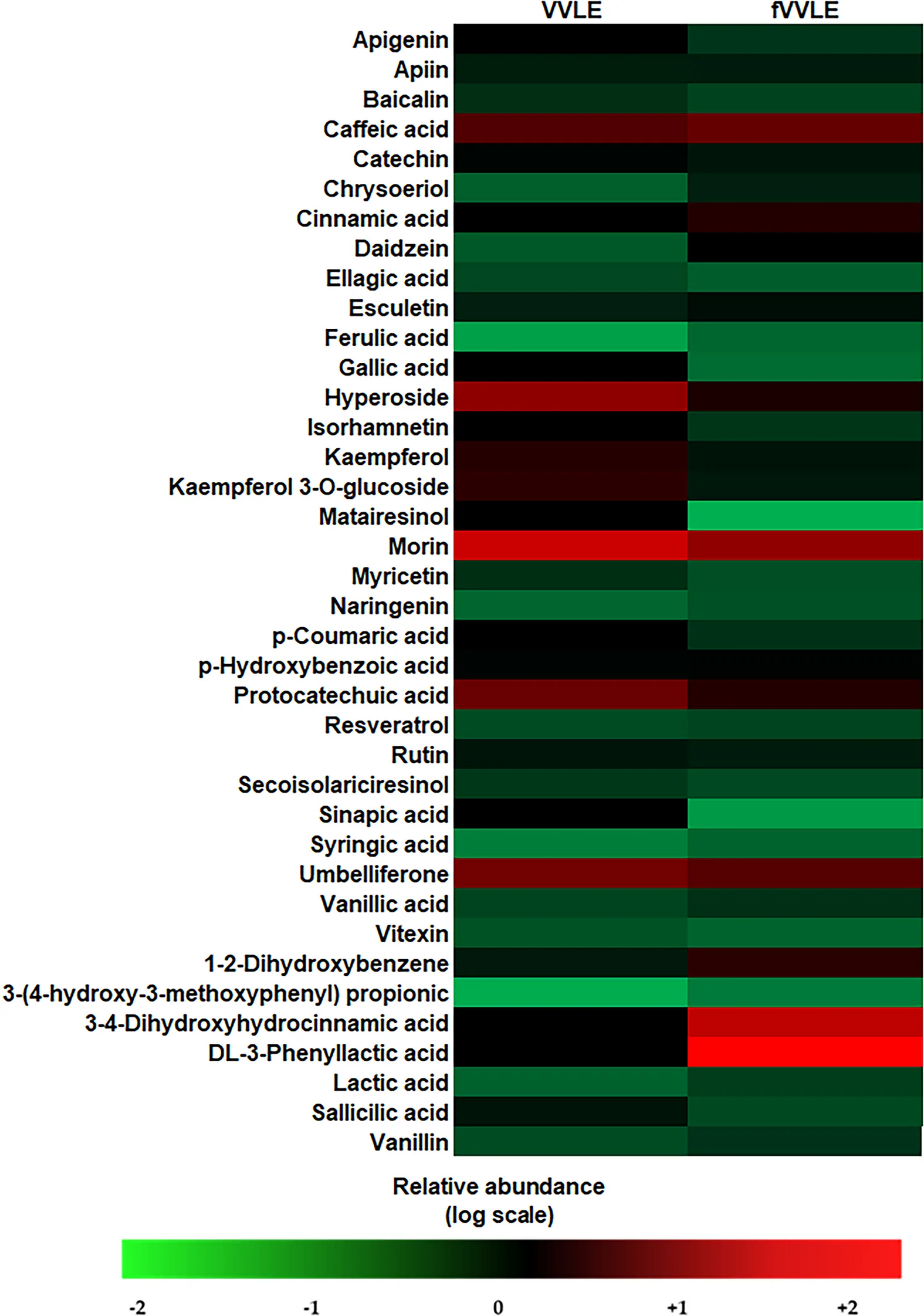
Metabolomic profiling of VVLE and fVVLE. VVLE, Vitis vinifera L. leaves extract; fVVLE, fermented Vitis vinifera L. leaves extract. Colors indicate the relative abundance (logarithmic scale) of compounds in each medium. Red and green represent high and low abundance, respectively

The comprehensive metabolic profiling enabled the annotation of 38 bioactive compounds included in the comparative analysis of the native grape extract and its 48-h fermented counterpart. The resulting heat map (Fig. 3) clearly highlighted distinct intensity patterns between the two samples, indicating an extensive rearrangement of the phytochemical content of grapevine leaf extract after lactic fermentation.

Compared with VVLE, fVVLE showed a clear depletion of several abundant grape flavonols and phenolic acids, including hyperoside (quercetin-3-O-galactoside), protocatechuic acid (PCA), gallic acid, kaempferol 3-O-glucoside and kaempferol, suggesting preferential microbial biotransformation and/or degradation during fermentation. In contrast, fermentation with *L. plantarum* led to the accumulation of hydroxycinnamic acid derivatives, phenolic acids, and simple phenols, some of which were absent or only in minor amounts in the starting extract, most notably 3,4-dihydroxyhydrocinnamic acid (DHCA), DL-3-phenyllactic acid (PLA), and 1,2-dihydroxybenzene (catechol). At the same time, several methoxylated hydroxycinnamic acids (i.e., ferulic, sinapic), benzoic derivatives (i.e., syringic, vanillic), ellagic acid and the lignan matairesinol exhibited marked negative log-fold changes, suggesting substantial microbial transformation and/or partial degradation of these higher-molecular-weight phenolics.

## Discussion

### Lactiplantibacillus plantarum PBS067 thrives in VVLE-supplemented medium

As reported by the ISAPP consensus on prebiotics, plant polyphenols meet the prebiotic criteria and act as such, thus shaping host health (Gibson et al. 2017; Hutkins et al. 2025; Petrov Ivanković et al. 2023). Furthermore, they may boost polyphenol-resistant bacteria, such as *Akkermansia muciniphila*, in the gut (Rodríguez-Daza et al. 2021). *L. plantarum* is a polyphenol-tolerant probiotic strain which can also easily adapt to different ecological niches, human gut included (Barroso et al. 2014; Echegaray et al. 2023; Özcan et al. 2021). All its features make it a good candidate for an in vitro probiotic-driven fermentation model for the exploitation of polyphenol-rich extracts. The first objective was the evaluation of the effects of VVLE on *L. plantarum* PBS067 in vitro growth, to assess whether the probiotic was positively or negatively affected by it. Our data suggest that *L. plantarum* PBS067 can adapt to VVLE-supplemented fermentation broth within a few hours as it accommodates the probiotic nutritional requirements (Popova-Krumova et al. 2024). Moreover, the starter well tolerates the presence of polyphenols, biotransforming them through its secondary metabolism. Lastly, based on these results we can hypothesize that the presence of several antibacterial phytoactives (e.g., kaempferol, gallic acid, salicylic acid, hyperoside) does not impair probiotic growth (Ramadan et al. 2017; Song et al. 2022; Sun et al. 2017; Tian et al. 2023).

### Fermentation with *L. plantarum* PBS067 remodels the phenolic metabolome of VVLE

Despite the extensive consensus that the phytochemical composition of *V. vinifera* by-products is significantly influenced by environmental factors (Gouvinhas et al. 2018; Tsoupras et al. 2023) and the extraction methodologies (Beilankouhi et al. 2024), the main classes of bioactive compounds identified in grapevine leaves are phenolic acids, flavonoids (mainly flavonols, flavan-3-ols and flavones), and stilbenes (Anđelković et al. 2015). According to previous evidence, our findings highlighted the presence of gallic acid, caffeic acid, kaemferol, quercetin derivatives, and resveratrol, among the others (Beilankouhi et al. 2024). The phenolic content of wine by-products is susceptible to considerable variations in chemical composition following bioprocessing, a phenomenon influenced by the selection of starters and the technological practices employed (Freitas et al. 2024; Garrido and Borges 2013).

In our study, flavonoid compounds were predominant in VVLE samples, whereas fermentation shifted the phytochemical profile towards phenolic acids, particularly hydroxycinnamic-derived acids and phenyllactic derivatives. The bioconversion of phenolic compounds was driven by *L. plantarum* PBS067 metabolism during the fermentation process. Indeed, the existing literature broadly demonstrates that complex phenolics can be converted into simpler derivatives by the esterase, decarboxylase, reductase, and glycosidase activity of *Lactobacillus* species (Gaur and Gänzle 2023; Nawaz et al. 2025). Among these, *L. plantarum* has been shown to metabolize polymeric hydroxybenzoic- and hydroxycinnamic-derived compounds in plant substrates via esterase and decarboxylase enzymes (Esteban-Torres et al. 2015; Muñoz et al. 2024, 2017; Wang et al. 2021). This could explain the increased abundance of DHCA observed in fVVLE: during fermentation, precursor compounds such as caffeic or chlorogenic acid, naturally present in VVLE, are likely to undergo hydrogenation of the C = C double bond in their side chains (Ricci et al. 2019). Similarly, the high level of catechol in fVVLE suggests a decarboxylation of dihydroxybenzoic acids, including PCA, mediated by *L. plantarum* (Jiménez et al. 2013; Landete et al. 2021). Furthermore, the intrinsic decarboxylase activity of the starter might explain the considerable reduction of gallic acid after the fermentation process, in agreement with previous evidence (Jiménez et al. 2013; Rodríguez et al. 2008). The synthesis of PLA in fVVLE has been previously observed from the *L. plantarum* catabolism of L-phenylalanine, a process that is extensively represented in *V. vinifera* (Ricci et al. 2019; Sharafan et al. 2023). In contrast, the depletion of hyperoside and kaempferol-3-glucoside is compatible with the glycosidic activity of the starter microorganism. According to our findings, Lee et al. reported that *L. plantarum*-mediated fermentation of plant materials rich in flavonoid glycosides leads to the release of the respective aglycone forms (Lee et al. 2015). Overall, the decrease in these native phenols indicates that *L. plantarum* employs a dynamic process of utilization and conversion of a variety of phenolic substrates, thereby altering the phytochemical composition of the grape extract. From a biochemical standpoint, these transformations may yield metabolites that could be more bioactive or bioavailable than the original forms (Muñoz et al. 2017).

### Lacto-fermentation slightly affects the total phenolic content of VVLE

The TPC analysis revealed that lacto-fermentation slightly increased the levels of most phenolic compounds in VVLE, although with no statistically significant differences between pre- and post-fermentation. This result is consistent with the existing literature, which has generally expressed concordant opinions about the positive correlation between *L. plantarum* fermentation and the increase in total phenolics (Ayar-Sümer et al. 2024; Hunaefi et al. 2012; Meng et al. 2022). Xiao et al. demonstrated that the TPC exhibited an increase during the fermentation process when soy whey was inoculated with *L. plantarum* B1-6 (Xiao et al. 2015). Nevertheless, the outcomes of the biotransformation mediated by starter cultures on polyphenols are recognized to be highly substrate- and strain-specific (Dikmetas et al. 2025; Markkinen et al. 2019; Ozturk et al. 2024; X. Zhao et al. 2023a, b). Several factors, including temperature, pH, fermentation time, cultivation environment and microorganism activity, have been shown to significantly influence the phytochemical composition of the grapevine by-products (Freitas et al. 2024; Z. Zhao et al. 2023a, b). Generally, the catalytic activity of LAB has been recognized as one of the main reasons for improving the bio-accessibility of polyphenolic compounds (Ozturk et al. 2024; Wu et al. 2021). In our *V. vinifera* leaf system, the modest variation in TPC may reflect the liberation of bound or glycosylated phenolic compounds, within their conversion into smaller, more reactive molecules by *L. plantarum* enzymes (Al Juhaimi et al. 2019). The increased trend in the phenolic content observed in fVVLE is consistent with the previously described metabolomics data, which demonstrated an accumulation of low-molecular-weight phenolics, including vanillic acid and DHCA, following the fermentation process. These changes have the potential to increase the bioactivity of the extract without causing a substantial alteration in the absolute amount of polyphenols, sufficient to be observed as a trend, but not enough to reach statistical significance.

### The antioxidant activity reduces throughout *L. plantarum* PBS067 fermentation

The fermentation-driven changes in grape leaf extract have important implications for the bioavailability of its bioactive compounds and their health-promoting properties (Singh et al. 2023; Sousa et al. 2024). Our results demonstrated a diminished antioxidant effect following *L. plantarum*-mediated biotransformation. Some of the phenolic compounds that decreased due to the bioprocessing, such as hyperoside and gallic acid, are widely recognized to be potent radical scavengers due to the presence of multiple hydroxyl groups on their aromatic rings (Hadidi et al. 2024; Liu et al. 2005; Shojaee et al. 2022; Sukito and Tachibana 2014). Despite the increase of catechol, an efficient DPPH quencher (Shimizu et al. 2010), the fermentation process concurrently enriched the matrix with additional compounds, including PLA and cinnamic acid. These aromatic acids have a significantly lower capacity to donate electrons due to their chemical structure (Moazzen et al. 2022; Suzuki et al. 2013). Consistent with our findings, Hunaefi et al. reported a drastic reduction in DPPH radical scavenging activity, directly corresponding to a loss of total phenolic content after 72-h *L. plantarum-*mediated fermentation (Hunaefi et al. 2012). It is reasonable to assume that, although fermentation releases some antioxidant derivatives, the total antiradical power of the extract decreases because the main high-potency phenols are bio-transformed or lost more rapidly than equally potent substitutes can accumulate.

*L. plantarum*-driven fermentation generates simpler phenol-derived acids along with other organic acids, mainly lactic acid, thus enhancing the antioxidant potential of LAB-fermented derivatives (Khubber et al. 2022; Yang et al. 2024). However, the resulting antioxidant activity strongly depends on the microenvironmental pH, which can affect both the stability and the dissociation rates of the functional groups of said compounds (Ghosh et al. 2015; Popova-Krumova et al. 2024).

Based on our results, it could be hypothesized that, despite the presence of both plant- and probiotic-derived antioxidant compounds, the decrease in the fVVLE antioxidant potential compared to the one of VVLE may be linked to the more neutral pH. At the same time, other factors (e.g., bioactive compounds, synergistic and/or antagonistic interactions) are not to be excluded yet.

### Impact of probiotic fermentation on protein content

The lactic fermentation led to a significant decrease in the total protein content compared to the original biomass, as quantified by the microBCA assay. Although high sample dilutions (1:500 and 1:1000) were systematically applied to mitigate matrix interferences, this sharp decline could be attributed to several factors. First, the chosen pH (between 5.8 and 6.4) establishes the ideal physiological window for the proteolytic machinery of *L. plantarum* (Giraud et al. 1991). This probiotic has a robust proteolytic system to satisfy its nitrogen requirements. Therefore, hydrolyzed plant proteins are able to support the LAB growth (Kieliszek et al. 2021), but they can directly impact the assay performance. The number of detectable copper-coordination sites drops drastically, and the spectrophotometric signal diminishes, despite the retention of nitrogen in the medium. Second, the observed protein concentrations are significantly affected by chemical interferences that cannot be eliminated despite the dilutions. In the pre-fermentation stage, there is a high baseline of bioactive polyphenols, like flavonoids and tannins. These compounds act as powerful reducing agents that directly reduce copper ions (Cu^2+^ to Cu^+^) within the assay, thereby artificially inflating the initial protein baseline (Singh et al. 2020). As the fermentation progresses, *L. plantarum* metabolizes or complexes these phenolics, potentially limiting this interference. Conversely, in the post-fermentation product, the co-presence of lactic acid and ammonium likely results in an acid-base neutralization reaction, creating a high concentration of ammonium lactate. Even after diluting it, the residual ammonium ions (NH^4+^), together with phenolic degradation products, can act as strong chelators, thus artificially underestimating the protein content.

Therefore, protein assessment of VVLE and fVVLE will require different experimental approaches, with more comprehensive analyses.

### Potential dermocosmetic applications of fVVLE

The presence of high levels of lactic acid, as quantified by its specific assay, and the chemical inferred enrichment in ammonium lactate make the fVVLE a valuable green ingredient with potential wide-scale applications. Indeed, both molecules are recognized for their beneficial dermatological properties. They work as moisturizing agents (especially in atopic dermatitis), improve epidermal proliferation and barrier, and exert keratinolytic effects in xerosis and psoriasis. Moreover, lactic acid additionally contributes antioxidant effects (Algiert-Zielińska et al. 2019; Emer et al. 2011; Feng et al. 2024). The unique combination of organic acids and corresponding salts, bioactive phenolics, and microbial-derived metabolites confers promising properties to the fermentation product, positioning it as a versatile ingredient for dermatological and cosmetics purposes. Beyond the above-mentioned benefits, the complex nature of fVVLE may positively modulate the host skin microbiota. As previously reviewed, the probiotic fermentation of grape leaf extracts potentially yields a synergistic blend of prebiotics and postbiotics that may directly enhance skin microbiota and health, although standardizing this bioprocess remains a key challenge (Armari et al. 2024). Although further studies are required to validate these effects and to standardize the fermentation process, the simultaneous targeting of epidermal barrier function and skin microbial balance suggests promising opportunities for the development of next generation dermocosmetic products based on upcycled grapevine biomass.

## Conclusion

In the present work, we investigated the effects of probiotic-driven fermentation of VVLE, an abundant plant waste. While the valorization of viticulture by-products, such as grape pomace, skins, or seeds, has been extensively explored, *Vitis vinifera* L. leaves have received remarkably poor attention. Notably, the biotechnological exploitation of such biomass is still largely underdeveloped despite its considerable potential. Although biotechnology frameworks allow precise and optimal fine-tuning of multiple parameters, extensive research is required to fully map and standardize these complex systems.

To address this significant gap in existing literary sources, the final goals of our research were to investigate the impact of a controlled pilot-scale in vitro fermentation of VVLE and to characterize both VVLE and fVVLE phenolic composition, antioxidant capacity, metabolomic profile, lactic acid production and protein content. Thus, we analyzed them by means of culture, pH shift, enzymatic, and metabolomics approaches.

Overall, the results presented here show how *L. plantarum* PBS067 is a good fermentation starter, as it thrives in the optimized fermentative conditions and in a polyphenol-rich medium. The selected strain proved remarkable tolerance to the phytoactives naturally present in grapevine leaves, successfully biotransforming complex phenolic compounds through its versatile enzymatic systems.

In addition, the fVVLE appears to have an interesting functional profile in terms of phenolic composition, although it has lost part of the original scavenging radical potential of the VVLE.

From a green standpoint, our research highlights a sustainable strategy to valorize viticulture by-products through precise lacto-fermentation while fitting within the circular economy model. Ultimately, the core novelty of this manuscript lies in establishing a pioneering, tightly controlled pilot-scale framework for a highly neglected botanical matrix, thereby expanding the frontiers of current viticulture upcycling beyond traditional boundaries.

Although further experiments are needed to investigate the potential of these ingredients in in vitro models, both prokaryotic (e.g., single/combined microbiological cultures or microbiome models) and eukaryotic, this study could pave the way for novel bioactive products with several possible applications, ranging from cosmetics, nutraceuticals, complementary medical uses, and pharmaceutical products.

## Supplementary Information

Below is the link to the electronic supplementary material.

ESM 1 (PDF 206 KB)

## Data Availability

The data supporting the findings of this study are available from the corresponding author upon reasonable request.
