# Supplementary material for: Effects of controlled fermentation by Lactiplantibacillus plantarum PBS067 on Vitis vinifera L. leaf extract
Source: Appl Microbiol Biotechnol. 2026 Jun 29;110:276. doi: 10.1007/s00253-026-13942-7 (PMC13578193; doi:10.1007/s00253-026-13942-7)

Effects of Controlled Fermentation by *Lactiplantibacillus plantarum* PBS067 on *Vitis vinifera* L. Leaf Extract

Marta Armari[1, 2, §], Federica Cascella[1, 2, §], Aurora Tritto[3], Farah Hamed[1, 2, #], Shwetha Bangalore Renukaprasad[1, 2, #], Ana Moreno Villena[4], Giuseppe Meca De Caro[4], Carlos Luz Mínguez[4], Cesar Francisco Trejo[3], Barbara Azzimonti[1, 2, 5, *]

AFFILIATIONS

[1] Department of Health Sciences, School of Medicine, Università del Piemonte Orientale, 28100 Novara, Italy; marta.armari@uniupo.it; federica.cascella@uniupo.it; 20064124@studenti.uniupo.it; 20061209@studenti.uniupo.it; barbara.azzimonti@uniupo.it

[2] Center for Translational Research on Autoimmune and Allergic Diseases, Università del Piemonte Orientale, 28100 Novara, Italy

[3] Roelmi HPC Srl, Via Celeste Milani 24/26, 21040 Origgio, Varese, Italy; aurora.tritto@roelmibiotech.com; cesar.trejo@roelmibiotech.com

[4] Laboratory of Food Chemistry and Toxicology, Faculty of Pharmacy, University of Valencia, Av. Vicent Andrés Estellés s/n, 46100 Burjassot, Spain; ana.moreno@uv.es; giuseppe.meca@uv.es; carlos.luz@uv.es

[5] Centro 3R - Centro Interuniversitario per la promozione dei principi delle 3R nella didattica e nella ricerca

[§] These Authors share co-first authorship

[#] These Authors equally contributed to the work

[*] Correspondence: barbara.azzimonti@uniupo.it; Tel +390321660870

SUPPLEMENTARY MATERIAL

Supplementary Table S1. *L. plantarum* PBS067 growth in Poly I medium during VVLE fermentation.

OD_{600} = optical density at 600 nm. Data are expressed as the mean ± SD

**Timepoints (T, hours)**	**OD_{600}**	**Log_{10} CFU/mL**
0	0 ± 0.00	7.83 ± 0.02
2	0 ± 0.00	7.85 ± 0.02
4	0.03 ± 0.02	8.37 ± 0.02
6	0.59 ± 0.00	9.32 ± 0.04
24	1.16 ± 0.00	10.12 ± 0.04
48	1.23 ± 0.03	9.07 ± 0.08

Supplementary Fig. S1. DPPH regression curve obtained from absorbance values at 517 nm of increasing dilutions of DPPH in 96% ethanol. Abs 517 nm: Absorbance at 517 nm; DPPH: 2,2-diphenyl-1-picrylhydrazyl. Data are expressed as mean ± SD.

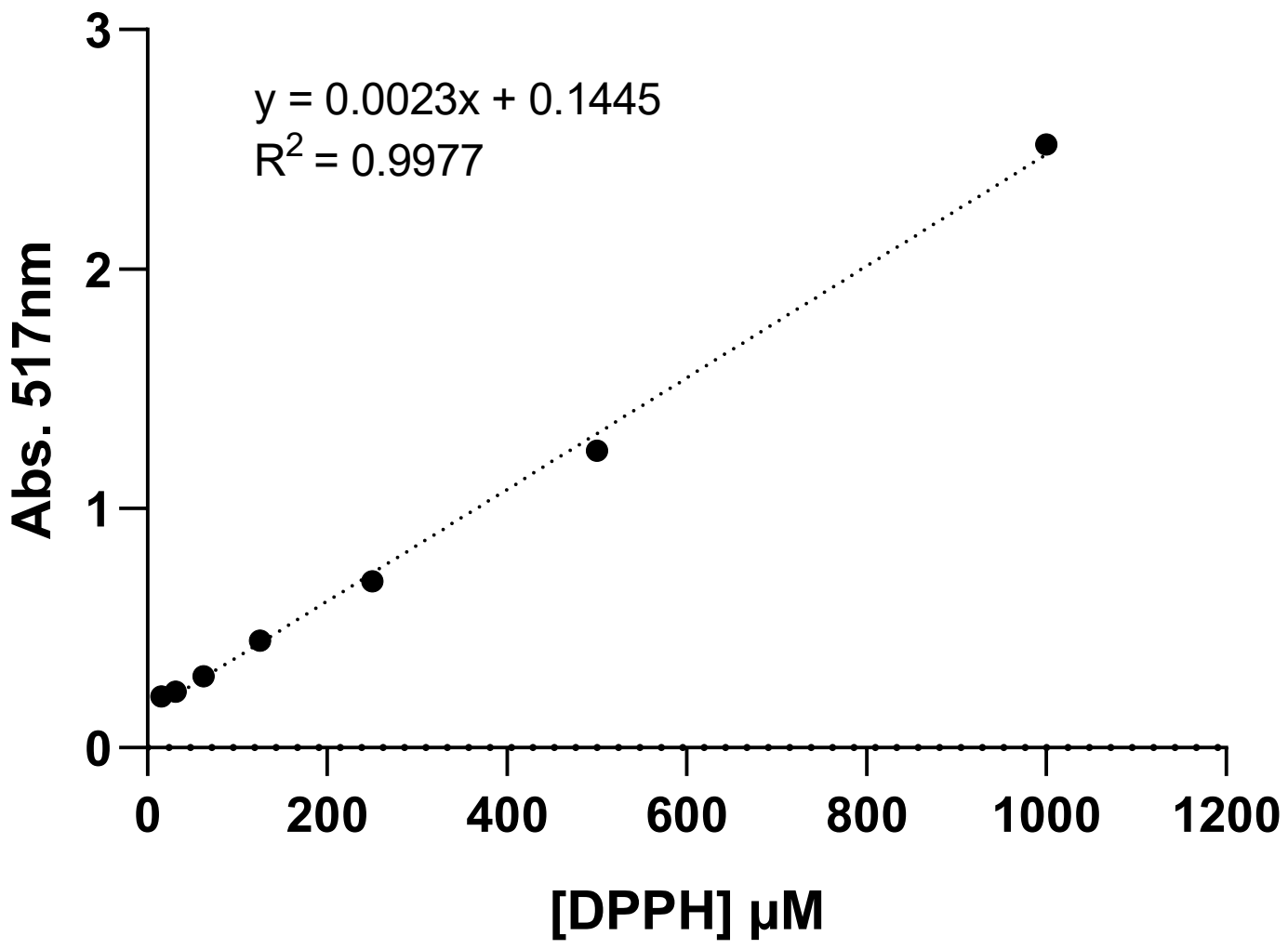

Supplementary Fig. S2. Gallic Acid regression curve obtained from absorbance values at 750nm. Abs 750: Absorbance at 750 nm. GA: gallic acid. Data are expressed as mean ± SD.

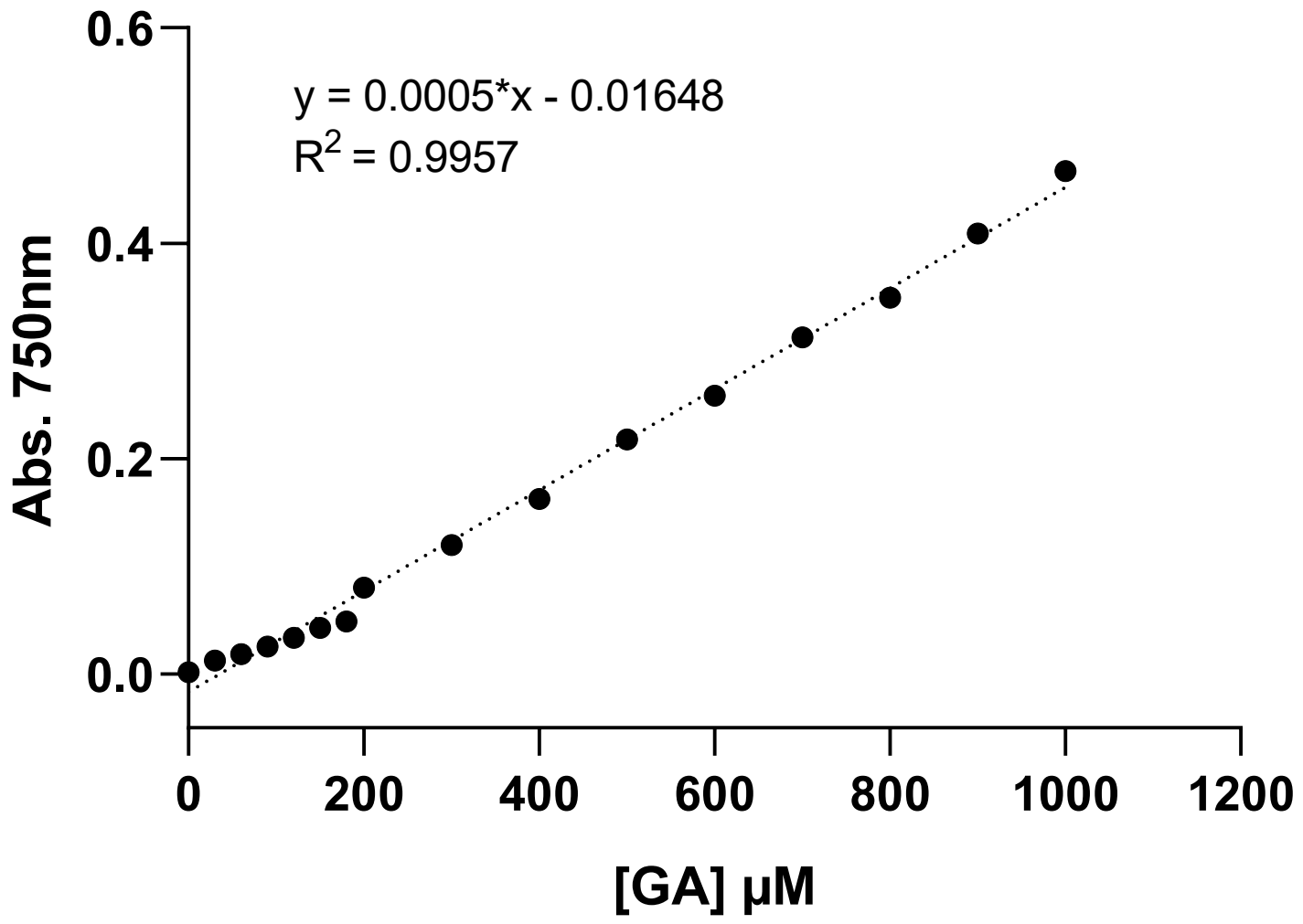

Supplementary Fig. S3. BSA (Bovine Serum Albumin) standard calibration curve, obtained through increasing dilutions of BSA in PBS 1X buffer, in a range of 0.5-200 µg/mL. Abs 562 nm: Absorbance at 562 nm. Data are expressed as mean ± SD.

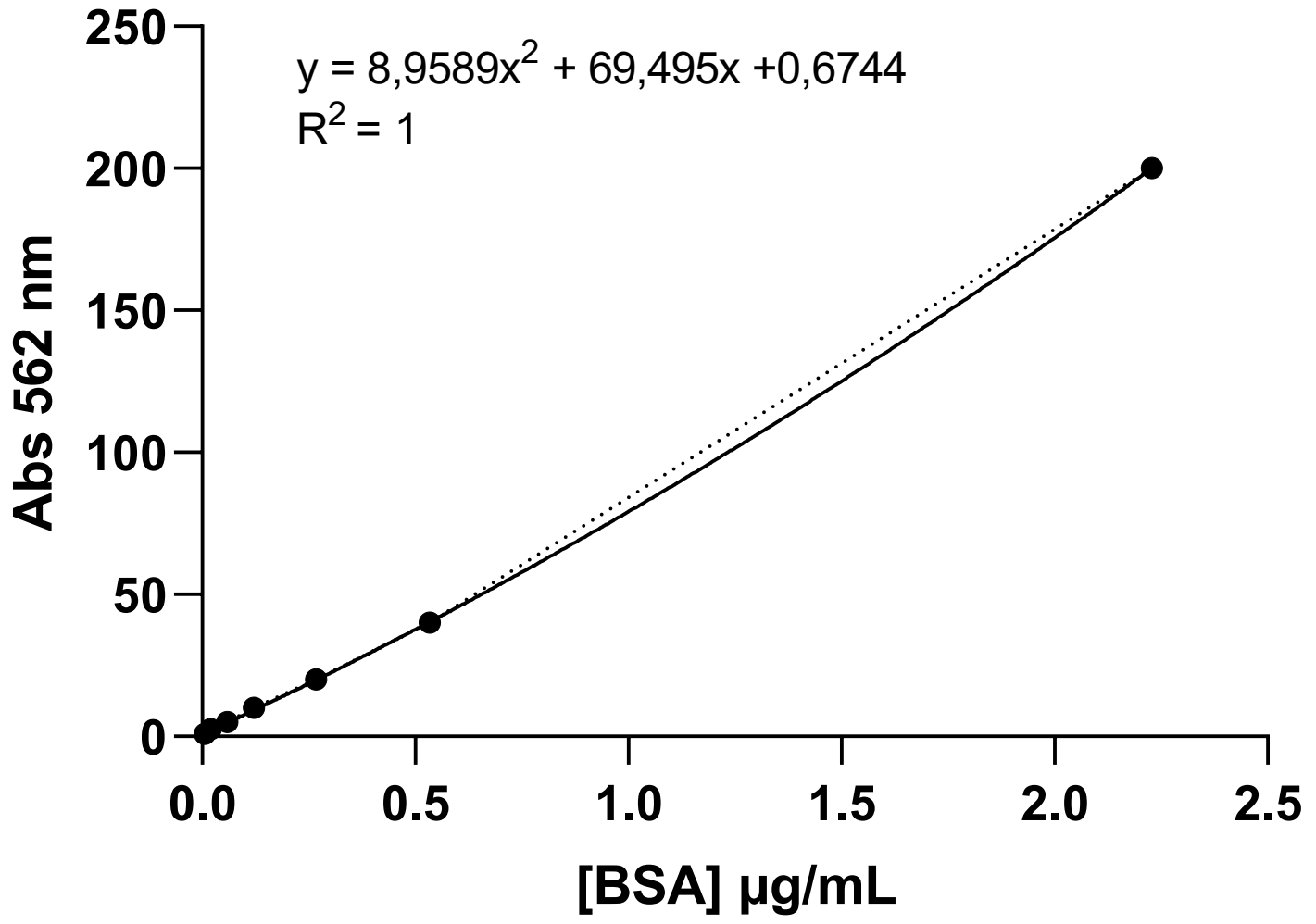

Supplement: Supplementary file 1 [file 253_2026_13942_MOESM1_ESM.pdf]
